# Alteration of monocyte subsets and their functions in thalassemia patients

**DOI:** 10.1007/s12185-022-03484-9

**Published:** 2022-11-02

**Authors:** Thanakrit Piyajaroenkij, Adisak Tantiworawit, Jeeraphong Khikhuntod, Pokpong Piriyakhuntorn, Thanawat Rattanathammethee, Sasinee Hantrakool, Chatree Chai-Adisaksopha, Ekarat Rattarittamrong, Lalita Norasetthada, Kanda Fanhchaksai, Pimlak Charoenkwan, Hathairat Thananchai

**Affiliations:** 1 Division of Hematology, Department of Internal Medicine, Faculty of Medicine, Chiang Mai University, 110 Intravaroros Road, A. Muang50200, Chiang Mai, Thailand; 2 Division of Hematology, Department of Internal Medicine, Faculty of Medicine, Ramathibodi Hospital, Mahidol University, Bangkok, Thailand; 3 Division of Immunology, Department of Microbiology, Faculty of Medicine, Chiang Mai University, Chiang Mai, Thailand; 4 Division of Hematology and Oncology, Department of Pediatrics, Faculty of Medicine, Chiang Mai University, Chiang Mai, Thailand

**Keywords:** Monocyte, TNF-α, Thalassemia, Transfusion-dependent thalassemia

## Abstract

Infection is one of the leading causes of mortality in thalassemia patients. This study aimed to examine qualitative and quantitative changes in monocytes in thalassemia patients. Monocytes were isolated from peripheral blood mononuclear cells and separated into subpopulations by flow cytometry. Cytokine levels were measured using quantitative real-time reverse transcription polymerase chain reaction (qRT-PCR) and sandwich enzyme-linked immunosorbent assay (ELISA). The primary endpoint was monocyte-derived TNF-α expression. A total of 78 patients and 26 controls were included. The mean log (TNF-α fold-change) by qRT-PCR was significantly lower in all thalassemia groups, at 1.27 in controls, versus 0.97 (*p* = 0.0014) in non-transfusion-dependent thalassemia (NTDT), 0.96 (*p* = 0.0004) in non-splenectomized transfusion-dependent thalassemia (TDT-NS), and 0.87 (*p* < 0.0001) in splenectomized transfusion-dependent thalassemia (TDT-S). Similarly, the mean 2-h TNF-α level measured by sandwich ELISA assay was significantly lower in all thalassemia groups, at 98.16 pg/mL in controls, versus 56.45 pg/mL (*p* = 0.0093) in NTDT, 39.05 pg/mL (*p* = 0.0001) in TDT-NS and 32.37 pg/mL (*p* < 0.0001) in TDT-S. Likewise, TDT patients had a significantly decreased percentage of non-classical monocytes, by approximately half compared to controls. Our results show that thalassemia major patients have clearly impaired monocyte counts and function.

## Introduction

Thalassemia is the most common disorder of hereditary hemolytic anemia worldwide, which is found at a high frequency and prevalence throughout the Mediterranean region, Africa, Southeast Asia, and Pacific regions including Thailand [1]. The prevalence of Hemoglobin H (HbH) and Hemoglobin H/Constant Spring (HbH/CS) disease is especially unique in the northern part of Thailand, reported as high as 1:65 of the general population [2]. Regular blood transfusions in combination with iron chelation are essential for the survival of thalassemia patients especially those with transfusion-dependent thalassemia (TDT). Inevitably, most patients suffer from various complications, such as pulmonary hypertension, cardiomyopathies, endocrinopathies, hypersplenism, hemochromatosis, and infection.

Infection is the second most common mortality cause in thalassemia patients with the prevalence varying from 22.5 to 66% [3]. Predisposing factors for infection in thalassemia patients include severe anemia, iron overload, splenectomy, and numerous defects of the immune system [3]. Retrospective data from the Thailand E-SAAN study have shown that iron overload is the predictive factor for the development of severe bacterial infection [4]. Various immunological defects as a result of iron overload have been observed in previous in vitro studies including changes in subpopulations of T lymphocytes by increasing cytotoxic T cells (CD8^+^), suppression and dysfunction of T helper cells (CD4^+^), reduce the activity of the complement system, impair immunoglobulin and inflammatory cytokines production, abnormalities in phagocytic activity of polymorphonuclear neutrophils (PMN) [3–5], and defects in lysosomal function in monocytes/macrophages [6].

Monocytes are innate immune cells derived and differentiated from myelo-monocytic stem cells. These cells are functionally heterogeneous characterized by the ability to phagocytose, produce cytokines, and also reactive oxygen species (ROS). Monocytes are classified into three subsets with distinct functions based on their CD14 and CD16 expression detected by monoclonal antibodies and flow cytometry [7]. In the major population, about 85% of total monocytes are classical monocytes (CD14^++^ CD16^−^), which play role in phagocytosis and IL-10 production, and are also involved in tissue repair. Non-classical (CD14^+^ CD16^++^) and intermediate (CD14^++^ CD16^+^) monocytes, 10% and 5% of the total respectively, are pro-inflammatory cytokines producer, such as TNF-α, IL-1β, IL-6, and IL-8 [7, 8]. Changes of the three subsets in different types of diseases, such as Crohn’s disease, Eales’s disease, Rheumatoid arthritis, and asthma, have been studied [7]. However, the relationship between monocyte subsets and their functions in patients with thalassemia has not been investigated.

## Materials and methods

### Study design and participants

We conducted a single-center, cross-sectional study at the Division of Hematology, Department of Internal Medicine, Chiang Mai University Hospital, Chiang Mai, Thailand from June 2019 to March 2020. We aimed to obtain qualitative and quantitative analyses of monocytes in thalassemia patients. We enrolled thalassemia patients aged from 18 to 60 years whose diagnosis was confirmed by hemoglobin analysis using a high-performance liquid chromatography (HPLC) method. We excluded patients with a history of recent or suspected active infections, congenital immunodeficiency disorders, and had recently received blood transfusion. Healthy individuals without comorbidities and active infection with similar age range were also enrolled to be the control group.

### Ethical approval and consent to participate

We obtained written informed consent from each participant or their guardian, consistent with the 1975 Declaration of Helsinki on Ethical Principles for Medical Research Involving Human Subjects. This study was approved by the ethical review board of the Faculty of Medicine, Chiang Mai University (STUDY CODE: Study code: MED-2561-05827), and all experiments were performed following relevant guidelines and regulations.

### Procedures

Eligible controls, and non-transfusion-dependent thalassemia (NTDT), non-splenectomized transfusion-dependent thalassemia (TDT-NS), and splenectomized transfusion-dependent thalassemia (TDT-S) patients were allocated in a 1:1:1:1 ratio. Current medications, including aspirin (ASA) [9], calcium supplements [10–12], vitamin D [10–12], vitamin B [10–12], and all iron chelators (oral, subcutaneous, and intravenous route administration) must be discontinued at least 7 days before the time of blood sample collection due to their effects on the immune cell functions.

A single fresh blood sample of 15 ml (mL) was drawn on the date of enrollment. Peripheral blood mononuclear cells (PBMCs) were isolated from the whole blood by density gradient centrifugation method using Lymphoprep™ reagent (AXIS-SHIELD Poc AS, Oslo, Norway). Cells were stained with Fluorescein isothiocyanate (FITC)-conjugated anti-CD14 (clone 61D3, Invitrogen, Thermo Fisher Scientific, Inc), Allophycocyanin (APC)-conjugated anti-CD16 (clone eBioCB16, Invitrogen, Thermo Fisher Scientific, Inc), and Phycoerythrin-Cyanin 5.1 (PC5)-conjugated anti-HLA-DR (clone Immu357, Immunotech, Beckman Coulter, France) to detect CD14, CD16, and HLA-DR respectively. Then, cells were analyzed by flow cytometer (FACSAria, BD Bioscience, US). Monocytes were gated based on their light scatter properties and 10,000 cells were acquired. The acquisition of flow cytometric data was analyzed by Kaluza Analysis Software 2.0 (Beckman Coulter, France). Monocytes were gated based on their forward scatter and side scatter properties. After that, CD14 versus HLA-DR gating was used to exclude NK cells (HLA-DR negative). Selected monocytes were plot between CD14 and CD16 to gate the monocyte subsets (classical: CD14^++^ CD16^−^, Intermediate: CD14^++^ CD16^+^, Non-classical: CD14^+^ CD16^++^). The remainder of each specimen was used to measure the serum ferritin levels on the date of the blood sample collection.

### Outcomes

The primary endpoint was monocyte-derived TNF-α expression. Briefly, monocytes were isolated from PBMCs using an adherence method. Then 2 × 10^5^ cells were stimulated with 100 mg/mL lipopolysaccharide (LPS) (L4516, Sigma, US). Since the kinetic of TNF-α mRNA and protein expression level of activated monocytes is different and with the limitation of monocyte numbers, the optimal activation time for detection of both TNF-α mRNA and protein in the same condition was used in this study. After two hours (2 h) of stimulation, cells were harvested, and culture supernatants were collected. The expression of TNF-α was measured using two different techniques. A quantitative real-time reverse transcription polymerase chain reaction (qRT-PCR) method was used to detect TNF-α messenger ribonucleic acid (mRNA) and sandwich enzyme-linked immuno-sorbent assay (ELISA) was used to measure the TNF-α level in the culture supernatant. The key secondary endpoint was the proportion of monocyte subsets identified by flow cytometry.

Total RNA was extracted from harvested cells using a NucleSpin^®^ RNA kit (Macherey–Nagel, Fisher Scientific, UK) and converted to cDNA using ReverTra Ace^®^ qPCR RT Master mix with gDNA Remover (Toyobo CO., LTD. Life Science Department, Osaka, Japan). The expression of TNF-α was evaluated using Thunderbird^®^ SYBR qPCR Mix (Toyobo CO., LTD. Life Science Department, Osaka, Japan) and Applied Biosystems^®^ 7500 Real-Time PCR System (Applied Biosystems; Thermo Scientific, Inc.). The following primer pairs were used: Forward, 5-CTTCTCGAACCCCGAGTGAC-3 and reverse 5-TGAGGTACAGGCCCTCTGATG-3 for TNF-α [13]; forward, 5-GAAGGTGAAGGTC GGAGTC-3 and reverse 5-GAAGATGGTGATGGGATTTC-3 for glyceraldehyde-3-phosphate dehydrogenase (GAPDH). The first step was pre-denaturation at 95^O^C for 60 s and followed by two steps of amplification, 95 °C for 15 s and 60 °C for 45 s, for 40 cycles. The expression level of TNF-α was normalized against the expression of GAPDH. The relative gene expression of TNF-α after stimulating monocytes with LPS was calculated using 2^−ΔΔCt^ method_,_ where ΔΔCT equal (*C*_*t*,TNF-α_ − *C*_*t*,GAPDH_)_LPS treatment_ – (*C*_*t*,TNF-α_ – *C*_*t*,GAPDH_)_mock control._

TNF-α level in the culture supernatant was measured using a Human TNF ELISA kit (HuTNF OptEIA™, BD Bioscience, US) according to the manufacturer’s instructions.

### Statistical analysis

Total sample size of 104 controls and thalassemia patients were needed to detect significantly expected 10% differences in the primary endpoint at the two-sided alpha level of 0.05 with 80% power. Clinical demographic and baseline characteristics of all participants and continuous variables were described in terms of mean with standard deviation (SD) or median (range). Categorical variables were reported using absolute values and frequencies (percentages). Total absolute monocyte counts (AMCs) were calculated by multiplying total white blood cell count with the percentage of total monocytes measured by an automated analyzer. In part of the absolute count of each monocyte subset, we calculated by multiplying total AMCs with percentage of each subset analyzed by flow cytometry. Analysis of primary outcome was performed using one-way Analysis of Variance (ANOVA) with Bonferroni correction to demonstrate the change in outcomes in terms of mean with SEM (standard error of the mean).

We performed univariate and multivariate analysis using a logistic regression model to define clinical predictive factors for infection and the effect of iron overload on monocyte functions after adjustment with age, gender, body mass index (BMI), and splenectomy status. The correlation between serum ferritin level and monocyte functions in thalassemia patients was analyzed by generalized linear regression model using Pearson correlation coefficients. A probability value of less than 0.05 was considered statistically significant. All statistical analyses were performed with IBM SPSS (Statistical Package for the Social Sciences) Statistics software, version 23.

## Results

### Patient characteristics

From June 2019 to May 2020, a total of 104 controls and thalassemia patients were enrolled and allocated into the 4 groups in 1:1:1:1 ratio (26 of each). Baseline healthy individual and patient characteristics were summarized in Table 1. The median age was 28 years (range 25–45) in controls, 51 years (range 21–60) in NTDT, 27 years (range 18–59) in TDT-NS, and 26 years (range 18–43) in TDT-S. Males were more predominant than females in all three thalassemia patient groups. HPLC hemoglobin analysis in 19 NTDT patients (73.1%) was HbH disease and in most TDT patients were Beta-thalassemia/Hemoglobin E disease (β^0^/β^E^). The median monocyte count was 5.4% in controls, 5.2% in NTDT, 4.3% in TDT-NS, and 8.2% in TDT-S, respectively. Nearly half of NTDT and all TDT patients had serum ferritin levels ≥ 1000 ng/ml and received iron chelation therapy. Approximately one-third of NTDT and TDT-NS and also half of the TDT-S patients had experienced infection resulting in hospitalization and six patients had laboratory confirmation of bacterial septicemia from at least two positive blood culture specimens. Detected causative organisms included *Enterococcus faecium* (*n* = 1), *Aeromonas hydrophila* (*n* = 1), *Campylobacter jejuni* (*n* = 1), *Escherichia coli* (*n* = 2), and *Streptococcus* spp. (*n* = 2).

**Table 1:** Patient characteristics

Patient characteristics (*N* = 104)	Control (*n* = 26)	NTDT (*n* = 26)	TDT-NS (*n* = 26)	TDT-S (*n* = 26)
Age, year (median, range)	28 (25–45)	51 (21–60)	27 (18–59)	26 (18–43)
*Sex, no. (%)*				
Male	15 (57.7)	21 (80.8)	18 (69.2)	17 (65.4)
Body mass index (BMI), kg/m^2^ (mean ± SD)	23.3 ± 3.6	20.9 ± 2.7	19.9 ± 2.1	19.1 ± 2.2
*Thalassaemia typing, no. (%)*				
HbH disease	NA	19 (73.1)	2 (7.7)	0
HbH Constant Spring (HbH/CS) disease	NA	4 (15.4)	0	0
Beta thalassaemia/Hemoglobin E (β^0^/β^E^)	NA	3 (11.5)	22 (84.6)	24 (92.3)
Homozygous beta thalassemia	NA	0	2 (7.7)	2 (7.7)
Hemoglobin, g/dL (mean ± SD)	14.3 ± 1.2	8.5 ± 1.8	7.3 ± 1.0	6.7 ± 1.2
Hematocrit, % (mean ± SD)	43.6 ± 2.8	28.4 ± 5.3	23.2 ± 3.3	21.5 ± 4.2
WBC count, cells/mm^3^ (mean ± SD)	5828.8 ± 1115.9	7913.3 ± 3888.1	7693.3 ± 2292.5	14,908.9 ± 3539.9
Corrected WBC count, cells/mm^3^ (mean ± SD)	5818.7 ± 1111.2	6183.1 ± 1963.8	6962.8 ± 2066.8	7104.7 ± 4037.3
Monocyte count, % (mean ± SD)	5.4 ± 1.2	5.2 ± 2.6	4.3 ± 1.2	8.2 ± 4.3
Classical monocyte	89.0 ± 4.6	89.1 ± 5.9	90.8 ± 3.5	92.5 ± 3.3
Intermediate monocyte	3.6 ± 1.6	5.4 ± 2.9	5.5 ± 2.6	3.6 ± 2.1
Non-classical monocyte	7.2 ± 3.9	5.5 ± 3.9	3.6 ± 2.6	3.8 ± 1.9
Serum ferritin, ng/mL (mean ± SD)	198.8 ± 138.7	1033.0 ± 963.2	2221.8 ± 1438.9	2681.48 ± 1946.8
*Serum ferritin range, no. (%)*				
≤ 800 ng/mL	26 (100.0)	14 (53.8)	3 (11.5)	3 (11.5)
801–1000 ng/mL	0	2 (7.7)	1 (3.9)	2 (7.7)
1001–2500 ng/mL	0	7 (26.9)	13 (50.0)	8 (30.8)
> 2500 ng/mL	0	3 (11.6)	9 (34.6)	13 (50.0)
No. of participants in receipt of iron chelation therapy, no. (%)	0	12 (50.0)	26 (100.0)	26 (100.0)
*Iron chelating agent(s), no. (%)*				
None	24 (100.0)	12 (50.0)	0	0
Deferoxamine	0	0	0	2 (7.7)
Deferiprone	0	11 (45.9)	13 (50.0)	9 (34.6)
Deferasirox	0	1 (4.2)	4 (15.3)	3 (11.5)
Deferoxamine + Deferiprone	0	0	6 (23.1)	7 (26.9)
Deferoxamine + Deferasirox	0	0	2 (7.7)	5 (19.3)
Deferiprone + Deferasirox	0	0	1 (3.9)	0
History of infection requiring hospitalization, no. (%)	0	7 (26.9)	8 (30.8)	13 (50.0)
History of infection > 1 episode/year, no. (%)	0	2 (7.7)	2 (7.7)	3 (11.5)
History of bacterial septicemia (confirmed by at least two positive blood culture specimens), no. (%)	0	1 (3.9)	1 (3.9)	4 (15.4)

BMI, Body Mass Index; HbH, Hemoglobin H disease; n, number; NA, not analyzed; NTDT, Non-transfusion-dependent thalassemia; SD, standard deviation; TDT-NS, non-splenectomized transfusion-dependent thalassemia; TDT-S, splenectomized transfusion-dependent thalassemia; WBC, white blood cell

## Outcomes

### Primary endpoint

**Fig. 1:**
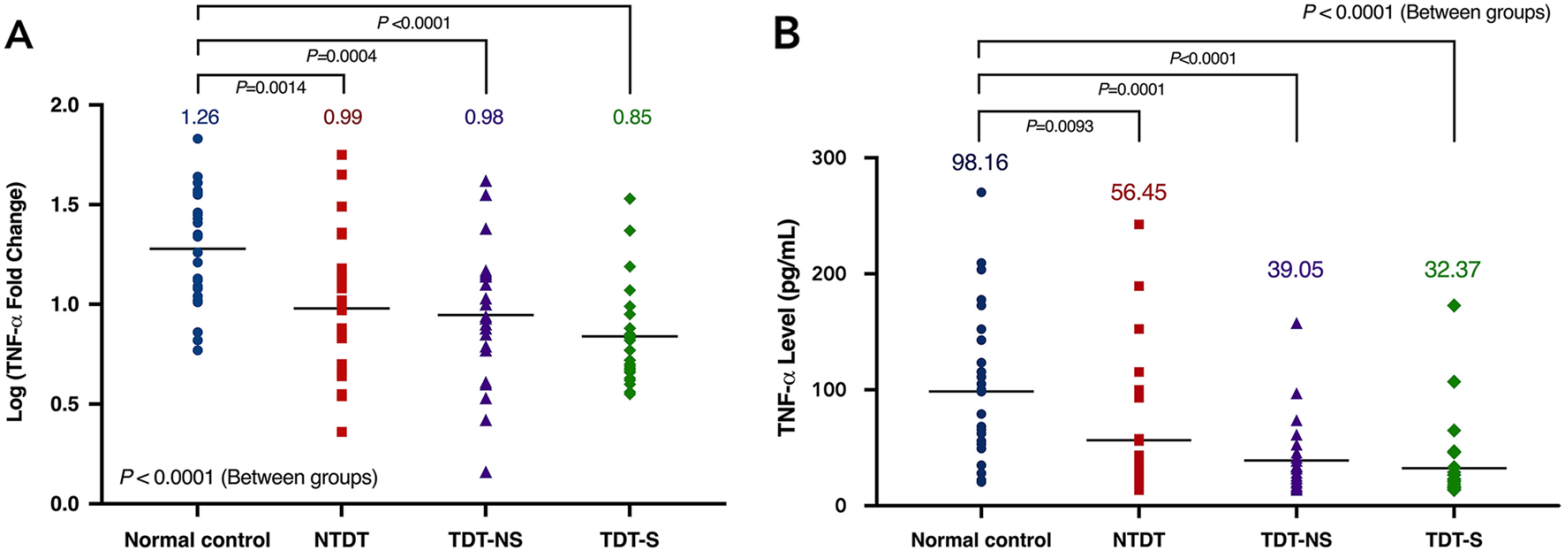
Monocyte-derived TNF-α expression. **a** Real-time RT-PCR method. **b** Sandwich ELISA assay

At the data cut-off date (30 April 2020), a total of 78 patients and 26 controls were included in the analysis. The 2-h monocyte-derived TNF-α expression fold-change after stimulation with LPS analyzed using qRT-PCR technique was significantly lower in all thalassemia groups with overall between-group *p* < 0.0001 (Fig. 1a). The mean log (TNF-α fold-change) was 1.26 (95% confidence interval (CI) 1.15–1.38) in controls, 0.99 (95% CI 0.80–1.17; *p* = 0.0014) in NTDT, 0.98 (95% CI 0.85–1.09; *p* = 0.0004) in TDT-NS and 0.85 (95% CI 0.76–0.96; *p* < 0.0001) in TDT-S. Similarly, the 2-h TNF-α level measured by sandwich ELISA assay was significantly decreased in all thalassemia groups with an overall between-groups *p* < 0.0001 (Fig. 1b). The mean TNF-α level was 98.16 pg/mL (95% CI 71.41–125.80) in controls, 56.45 pg/mL (95% CI 32.64–80.25; *p* = 0.0093) in NTDT, 39.05 pg/mL (95% CI 26.49–51.61; *p* = 0.0001) in TDT-NS and 32.37 pg/mL (95% CI 18.16 to 46.58; *p* < 0.0001) in TDT-S, respectively (Table 2).

**Table 2:** Monocyte-derived TNF-α expression

Results	Control (*n* = 26)	NTDT (*n* = 26)	TDT-NS (*n* = 26)	TDT-S (*n* = 26)
TNF-α fold-change, mean (95% CI)	22.71 (17.02–29.88)	14.79 (8.58–22.22)	12.17 (8.37–16.71)	8.59 (6.17–11.86)
Log(TNF-α fold-change), mean (95% CI)	1.26 (1.15–1.38)	0.99 (0.80–1.17)	0.98 (0.85–1.09)	0.85 (0.76–0.96)
Pre-stimulated TNF-α level, pg/mL (mean; 95% CI)	0.80 (0.54–1.07)	0.94 (0.68–1.19)	1.29 (1.02–1.56)	0.98 (0.73–1.24)
Post-stimulated TNF-α level, pg/mL (mean; 95% CI)	98.16 (71.41–125.80)	56.45 (32.64–80.25)	39.05 (26.49–51.61)	32.37 (18.16–46.58)

CI, confidence interval; TNF-α, tumor necrosis factor α

### Secondary endpoints

**Fig. 2:**
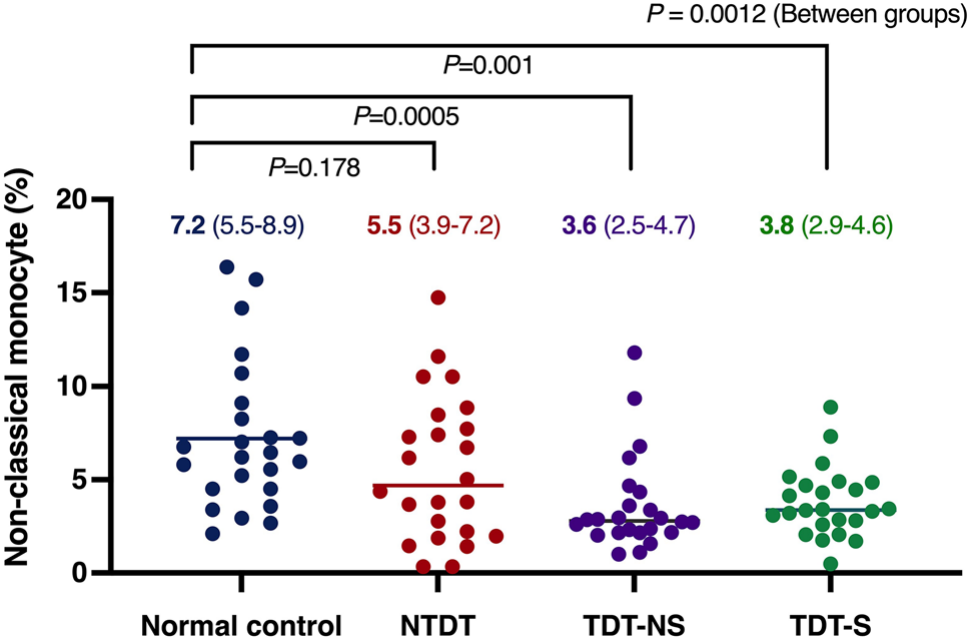
Non-classical monocyte subpopulation by flow cytometry

Results of the flow cytometric analysis of non-classical monocyte subpopulations show a concordant correlation to TNF-α expression. Statistically significant lowered numbers of non-classical monocytes were observed in the thalassemia major groups both NTDT and TDT with an overall between-group *p* = 0.0012 (Fig. 2). The mean non-classical monocyte numbers were 7.2% (95% CI 5.5–8.9) in controls, 5.5% (95% CI 3.9–7.2; *p* = 0.178) in NTDT, 3.6% (95% CI 2.5–4.7; *p* = 0.0005) in TDT-NS and 3.8% (95% CI 2.9–4.6; *p* = 0.001) in TDT-S. The absolute number of monocytes and their subsets is shown in Table 3.

**Table 3:** Absolute number of monocytes and their subsets

Results	Control (*n* = 26)	NTDT (*n* = 26)	TDT-NS (*n* = 26)	TDT-S (*n* = 26)
Absolute monocyte count, cells/mm^3^ (mean ± SD)	309.8 ± 93.6	351.0 ± 156.5	278.1 ± 104.5	512.7 ± 346.8
Classical monocyte	273.5 ± 79.6	310.9 ± 150.5	251.4 ± 99.1	476.7 ± 324.9
Intermediate monocyte	13.1 ± 9.7	20.9 ± 11.2	16.2 ± 8.2	17.8 ± 13.3
Non-classical monocyte	23.1 ± 18.4	21.2 ± 17.6	10.4 ± 7.9	18.3 ± 16.8

**Fig. 3:**
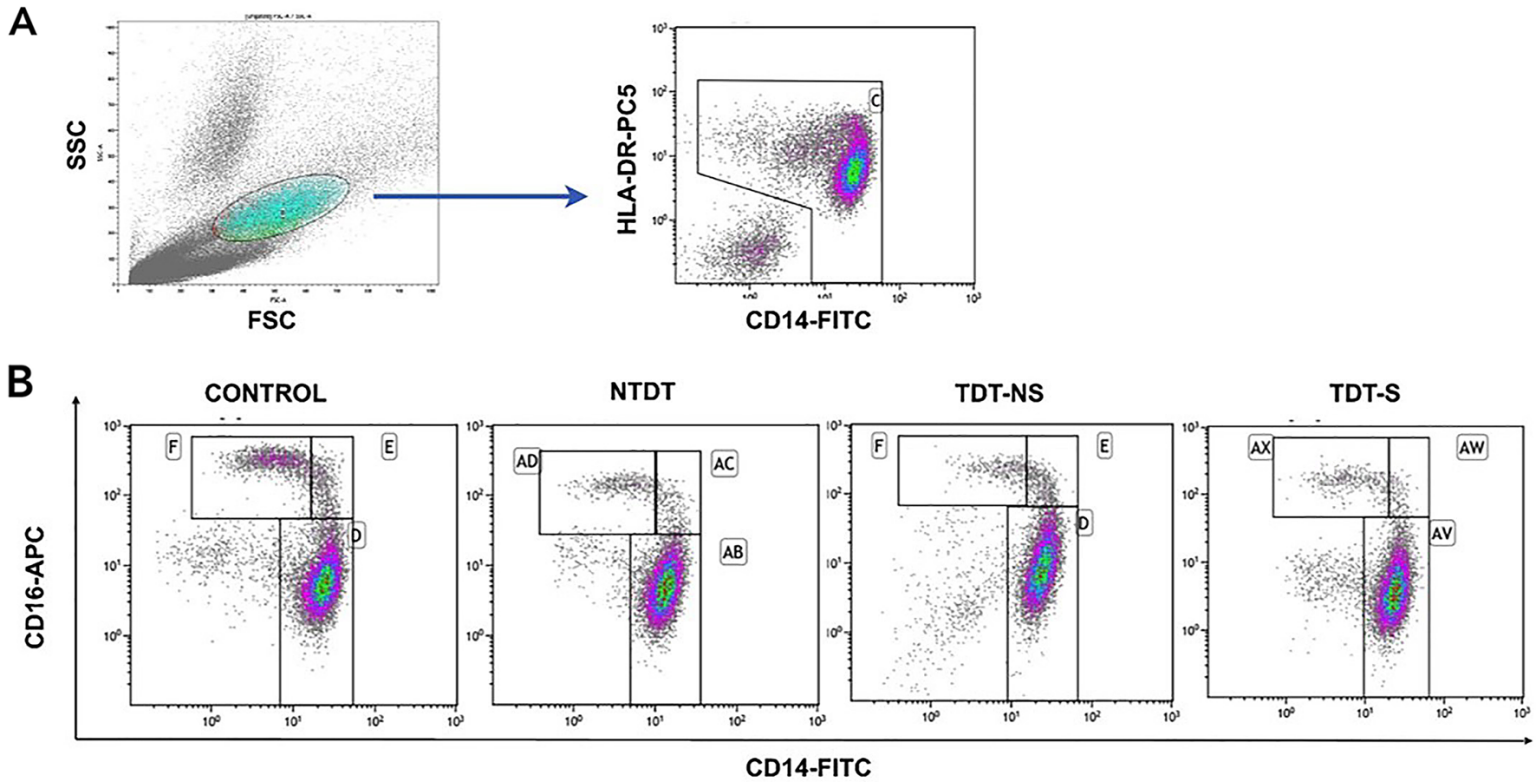
Monocyte subpopulations by flow cytometry

Gating strategy of the three monocyte subpopulations in PBMCs. (A) Monocytes were gated based on their forward scatter and side scatter properties. After that, CD14 versus HLA-DR (Human leukocyte antigen DR isotype) gating was used to exclude NK (Natural killer) cells (HLA-DR negative). (B) Selected monocytes were plotted between CD14 and CD16 to gate the monocyte subsets (classical: CD14^++^ CD16^−^, Intermediate: CD14^++^ CD16^+^, Non-classical: CD14^+^ CD16^++^). Each panel represents monocyte subpopulations from each study group (Fig. 3).

### Relationship between serum ferritin level, monocytic functions, and infection in thalassemia patient

The excessively high serum ferritin levels obtained on the day of blood sample collection were approximately fivefold in NTDT, tenfold in TDT-NS, and 12-fold higher than observed in the control group, respectively (Table 1). Hyperferritinemia may reflect the iron load burden in thalassemia patients.

**Fig. 4:**
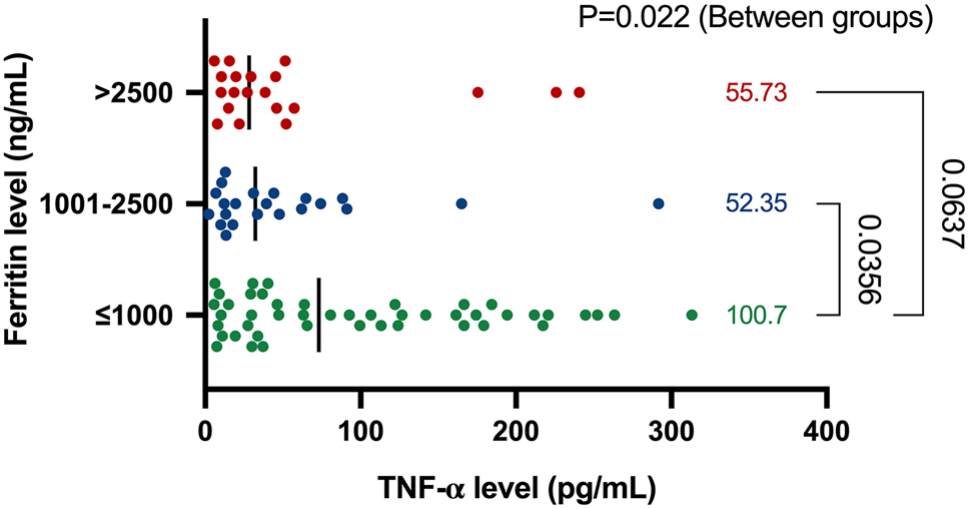
Monocyte-derived TNF-α expression in different serum ferritin range

The mean TNF-α levels were 100.7 pg/mL (95% CI 75.5–125.9) in patients with serum ferritin ≤ 1000 ng/mL, 52.4 pg/mL (95% CI 23.1–81.6) in patients with serum ferritin 1000 to 2500 ng/mL and 55.7 pg/mL (95% CI 22.5–88.9) in patients with serum ferritin > 2500 ng/mL with overall between-group *p* = 0.0022 (Fig. 4).

When comparing thalassemia patients with infection to those without infection, the mean serum ferritin levels were higher in the TDT-NS group with experienced infection but showed no statistical significance, 3463 pg/mL (95% CI 1607–6589) and 2497 pg/mL (95% CI 1481–3518; *p* = 0.63). Also, the mean serum TNF-α after 2-h LPS activation was lower in infected patients although no statistical significance (Table 4).

**Table 4:** Serum ferritin level and TNF-α level after LPS stimulation in thalassemia patients with and without bacterial infection

Results	NTDT (*n* = 26)		TDT-NS (*n* = 26)		TDT-S (*n* = 26)	
	Infection (*n* = 7)	No infection (*n* = 19)	Infection (*n* = 8)	No infection (*n* = 18)	Infection (*n* = 13)	No infection (*n* = 13)
Ferritin level, ng/mL (mean; 95% CI)	1277 (501–2292)	1486 (589–2551)	3463 (1607–6589)	2497 (1481–3518)	2495 (1414–3739)	3569 (2011–5161)
*p* value	0.82		0.63		0.34	
Post-stimulated TNF-α level, pg/mL (mean; 95% CI)	84.1 (18.7–164.3)	79.1 (31.7–140.6)	29.2 (14.6–44.9)	92.8 (29.7–162.5)	22.4 (9.3–35.2)	62.6 (22.6–109.9)
*p* value	0.86		0.13		0.24	

The univariate logistic regression analysis of risk factors for infection in patients with thalassemia is shown in Table 5. All analyzed clinical predictive factors showed no statistical significance for infection.

**Table 5:** Univariate analysis of risk factors of infection in 78 thalassemia patients

Variables	Number (*n* = 78)	% of infection (*n* = 28)	OR	95% CI	*p* value
*Age*					
< 40 years	55	72.4	0.76	0.23–2.52	0.66
≥ 40 years	23	27.6	1	–	–
*Gender*					
Female	55	62.1	2.38	0.79–7.11	0.12
Male	23	37.9	1	–	–
*Splenectomy*					
Yes	26	60.7	2.20	0.63–7.70	0.22
No	52	39.3	1	–	–
*Hemoglobin ≤ 7 g/dL*					
Yes	32	48.3	0.80	0.27–2.35	0.69
No	46	51.7	1	–	–
*Serum ferritin > 1000 ng/mL*					
Yes	54	69.0	0.64	0.17–2.48	0.52
No	24	31.0	1	–	–
*Serum ferritin > 2500 ng/mL*					
Yes	25	27.6	2.38	0.61–9.25	0.21
No	53	72.4	1	–	–
*Deferoxamine therapy*					
Yes	21	24.1	1.57	0.40–6.10	0.52
No	57	75.9	1	–	–
*Deferiprone therapy*					
Yes	45	55.2	1.78	0.57–5.60	0.32
No	33	44.8	1	1	–

However, we used a generalized linear regression model to analyze the effect of high serum ferritin levels on monocyte-derived TNF-α secretion efficacy after adjustment with other covariates, such as age, gender, BMI, and splenectomy status. Our results indicated that a cut-off level of serum ferritin higher than 1000 ng/mL was significantly associated with a decreased efficacy of TNF-α expression estimated 5.9-fold (95% CI −11.43 to −0.44; *p* = 0.034) compared to serum ferritin level less than 1000 ng/mL.

## Discussion

Blood monocytes are one component of the human mononuclear phagocyte system sharing features with macrophages and conventional dendritic cells, which play an important role in immune defense, inflammation, and homeostasis by clearing pathogenic organisms and dead cells, and also contribute to the tissue repairing process [14]. Based on surface CD14 and CD16 immunostaining, monocytes can be categorized into three subsets with distinct functions [7]. Importantly, intermediate and non-classical monocytes have responded to different types of bacterial [15], mycobacterial [16], and viral infections, such as hepatitis B virus (HBV) [17], hepatitis C virus (HCV) [18] and human immunodeficiency virus (HIV) [19]. Monocytic dysfunction and perturbation of their subsets in different types of autoimmune and inflammatory diseases are found in previous studies [7]. However, there are limited published data regarding the relationships of monocyte subsets and their functions in thalassemia patients.

Our findings demonstrated that monocyte-derived TNF-α production by qRT-PCR and ELISA techniques in thalassemia patients was significantly lower than that in healthy controls (overall between-group *p* < 0.0001); in response to LPS approximately 1.3–1.7-fold in NTDT, 1.3–2.5-fold in TDT-NS and 1.5–3-fold in TDT-S, which confirmed to earlier result. A recent study by Ud-naen et al. [20] compared the pattern of cytokine production in human monocytes from normal individuals and thalassemia patients in response to *Pythium insidiosum* zoospore exposure. The results showed that basal levels of granulocyte–macrophage colony-stimulating factor (GM-CSF) and also TNF-α production in thalassemia patients were significantly lower than non-thalassemia individuals.

As mentioned earlier, the major subset of circulating monocytes up to 85% is classical monocytes. Non-classical and intermediate monocytes are found in smaller numbers, approximately 10 and 5% of the total, respectively [7]. Similarly, the mean (SD) of monocyte subpopulations in the control group were 89.0% (4.6%) (classical), 7.2% (3.9%) (non-classical), and 3.6% (1.6%) (intermediate) in our study. In thalassemia patients, the percentage of non-classical monocyte subpopulation also decreased significantly by about twofold in TDT-NS and TDT-S compared to controls.

Some microbial pathogens need iron from healthy hosts for its survival. Among such organisms are *Yersinia enterocolitica*, *Klebsiella* species, *Escherichia coli*, *Streptococcus pneumoniae*, *Pseudomonas aeruginosa*, *Listeria monocytogenes*, and *Legionella pneumophila* have increased virulence in vitro in the presence of excessive iron load [21]. Our study found 4 of 9 patients had positive blood culture with *Escherichia coli* and *Streptococcus* spp.

Long-term regular blood transfusion is possible cause of immune dysfunction, called transfusion-induced immunomodulation (TRIM). Indeed, chronic allo-antigenic stimulation by residual donor antigen-presenting cells (APCs) following repeated transfusions induce T cell anergy, shifts toward T helper-2 (Th2) CD4^+^ T cell immunosuppressive phenotype, downregulate IL-2, IL-12 production, and leads to express numerous immunosuppressive cytokines, such as IL-4, IL-5, IL-10. This resulted in impairment of immunity including monocyte activation and cytocidal activity [22]. Recently, Vinchi et al. [23] found that transfusion led to iron-overload-mediated toxicity in macrophages/monocytes and also blunted their inflammatory response to infectious stimuli by increasing IL-10 production.

Splenectomy is another risk factor related to changes in immune responses in thalassemia patients [3]. Post-splenectomized thalassemia major patients have lower monocyte–macrophage numbers, and reduce immuno-globin M (IgM) level, complement system activation, and CD4/CD8 lymphocyte ratio in comparison to non-splenectomized patients [24]. Absolute numbers of lymphocyte are increased, the percentage of B lymphocytes is higher, although the percentage of T cells is reduced. Teawtrakul et al. [4] found that splenectomy > 10 years in splenectomized NTDT was a potential predictive factor for severe bacterial infection. The univariate analysis of risk factors for infection from our study revealed higher rate of infection was found in splenectomized thalassemia patients with odds ratio of 2.20 but no statistical significance due to small number of events.

Our study has some limitations. This is a single-center study initiated in a northern part of Thailand and all participants are Asian. Many studies have shown that variation in monocytes can be influenced by ethnicity [25]. European populations tend to have a higher monocyte count compared to African American and Japanese individuals [26]. In addition, Caucasian populations tend to have a lower number of non-classical monocytes than African populations [27]. Therefore, the findings from this study may be limited to apply in non-Asian populations. In addition, there are some differences in baseline characteristics between the groups. As regards, male patients are more frequent in all three thalassemia groups and the median age in the NTDT group is greater than other groups. Fewer monocyte subset differences in advanced age (80–100 years) have been reported [28]. However, none in this study is aged greater than 60 years. These limitations may not likely have a significant impact on our results. Concerning about gender, Tollerud et al. [29] carried out a population-based study to investigate the impact of age and gender on the cellular immune system. The results showed that age and/or gender had no effect on CD14^+^ or CD16^+^ monocyte numbers and functions.

## Conclusion

Our results show that thalassemia patients, especially those with TDT, have impaired numbers and functions of monocytes. The results also show an inverse effect of hyperferritinemia on cytokine production by monocytes. This may contribute to immunological impairment in patients with thalassemia and increase in the susceptible risk of infection.

## Data Availability

The data sets used and/or analyzed during the current research are available from the corresponding author on reasonable request.

## Abbreviations

ANOVA: A one-way analysis of variance
APC: Allophycocyanin
APCs: Antigen-presenting cells
ASA: Aspirin
BCECF: 2′,7′-Bis-(Carboxyethyl)-5-(and-6)-carboxyfluorescein
BMI: Body mass index
CAD: Coronary artery disease
CD: Cluster of differentiation
cDNA: Complementary deoxyribonucleic acid
ELISA: Enzyme-linked immunosorbent assay
FITC: Fluorescein isothiocyanate
FSC: Forward scatter
GAPDH: Glyceraldehyde-3-phosphate dehydrogenase
GM-CSF: Granulocyte–macrophage colony-stimulating factor
HbH: Hemoglobin H disease
HBV: Hepatitis B virus
HCV: Hepatitis C virus
HIV: Human immunodeficiency virus
HLA-DR: Human leukocyte antigen DR isotype
HPLC: High-performance liquid chromatography
Ig: Immunoglobulin
IL: Interleukin
LPS: Lipopolysaccharide
mRNA: Messenger ribonucleic acid
MOI: Multiplicity of infection
NA: Not analyzed
NK: Natural killer
NTDT: Non-transfusion-dependent thalassemia
PBMCs: Peripheral blood mononuclear cells
PC5: Phycoerythrin-Cyanin 5.1
PE: Phycoerythrin
qRT-PCR: Quantitative real-time reverse transcription polymerase chain reaction
SD: Standard deviation
SEM: Standard error of the mean
SPSS: Statistical Package for the Social Sciences
SSC: Side scatter
TCR: T cell receptor
TDM: THP-1 (human monocytic cell line)-derived macrophage
TDT: Transfusion-dependent thalassemia
TDT-NS: Non-splenectomized transfusion-dependent thalassemia
TDT-S: Splenectomized transfusion-dependent thalassemia
Th2: T helper-2 CD4^+^ T cell
TNF: Tumor necrosis factor
TRIM: Transfusion-induced immunomodulation
WBC: White blood cell
